# Keratinizing squamous cell carcinoma of the distal Esophagus in a young adult woman with celiac disease: a case report

**DOI:** 10.1093/omcr/omag178

**Published:** 2026-10-04

**Authors:** Samiullah Shaikh, Sidra Samad, Muhammad Saad, Areeba Ali Khan, Faizan Shaikh, Riaz Ahmed, Kamil Ahmad Kamil

**Affiliations:** Indiana University School of Medicine, Internal Medicine Department, 2651 E Discovery Pkwy, Bloomington, IN, 47401, United States; Liaquat University of Medical and Health Sciences, Jamshoro, Pakistan; Liaquat University of Medical and Health Sciences, Jamshoro, Pakistan; Services Institute of Medical Sciences, Lahore, Pakistan; Indiana University Health Bloomington Hospital, Bloomington, IN, United States; Liaquat University of Medical and Health Sciences, Jamshoro, Pakistan; Mirwais Regional Hospital, Kandahar 3801, Afghanistan

**Keywords:** Esophageal squamous cell carcinoma, celiac disease, oncology, gastroenterology, case report

## Abstract

Esophageal squamous cell carcinoma (ESCC) usually affects the upper or middle esophagus and is strongly associated with tobacco and alcohol use. We report a 28-year-old woman with longstanding celiac disease who presented with progressive dysphagia, weight loss, retrosternal pain, and melena. Endoscopy showed a partially obstructing distal esophageal mass; biopsy confirmed moderately differentiated keratinizing ESCC, while duodenal biopsy showed active celiac disease. CT and PET imaging revealed regional lymphadenopathy without distant metastasis. She underwent feeding jejunostomy, nutritional support, and neoadjuvant chemoradiotherapy, but follow-up imaging showed interval disease progression. The patient’s young age, squamous cell histology in a distal esophagus, and the absence of conventional risk factors make this an unusual presentation. This case emphasizes the need for high clinical suspicion and early endoscopic evaluation in young patients with celiac disease presenting with alarming symptoms, even in the absence of classic risk factors for esophageal cancer.

## Introduction

Esophageal carcinoma is the seventh most common malignancy worldwide and the sixth leading cause of cancer-related deaths, accounting for approximately 544 000 deaths in 2020 [1]. More than 90% of esophageal cancers are either esophageal squamous cell carcinomas (ESCC) or esophageal adenocarcinomas (EAC) [2]. ESCC predominantly affects the upper and middle thirds of the esophagus and is more common in men. Established risk factors for ESCC include tobacco and alcohol use, achalasia, caustic ingestion, tylosis, poverty, frequent consumption of hot beverages, and a history of head and neck cancer or chest irradiation [3].

Celiac disease (CD) is a chronic autoimmune enteropathy characterized by mucosal inflammation and villous atrophy, and has been associated with an increased risk of several malignancies, including esophageal cancer [4]. Moreover, in Pakistan, the mean age at ESCC diagnosis is approximately 49 years [5]. We report a rare case of distal keratinizing ESCC in a 28-year-old woman with longstanding celiac disease and no conventional ESCC risk factors, and to our knowledge, such a case has not previously been reported. This case was described in accordance with the CARE guidelines [6].

## Case report

A 28-year-old Pakistani woman presented with a three-month history of progressive mechanical dysphagia, initially to solids and later to liquids. This was associated with odynophagia, heartburn, and retrosternal pain radiating to the left side of the chest. She also reported two to three episodes of melena in the preceding week and an unintended weight loss of about 8 kg over the last six months. She denied fever, drooling, or dysarthria.

Her history was significant for childhood-onset steatorrhea and intermittent abdominal discomfort, diagnosed as celiac disease in childhood, with symptomatic improvement on a gluten-free diet. She had received five blood transfusions since the age of eight. There was no family history of gastrointestinal malignancy.

On examination, she appeared frail, with a BMI of 16 kg/m^2^ and conjunctival pallor. Vital signs were stable. Abdominal examination showed mild epigastric fullness without organomegaly. No peripheral lymphadenopathy was noted, and other systemic examinations were unremarkable.

Laboratory investigations (Table 1) revealed microcytic hypochromic anemia and hypoalbuminemia. Serology showed a positive anti-tissue transglutaminase IgG antibody level of 22.8 U/mL, borderline low anti-TTG IgA level of 1.6 U/mL, and markedly reduced total serum IgA level of 4 mg/dL, consistent with celiac disease in the setting of selective IgA deficiency.

**Table 1:** Laboratory results of the patient.

Parameter	Result	Normal range
**Complete Blood Count**		
Hemoglobin	7.5 g/dL	12.0–16.0 g/dL
Hematocrit	23%	36%–46%
RBC	3.86 × 10^12^/L	3.5–5 × 10^12^/L
MCV	72.7 fL	76–96 fL
MCHC	32.8 g/dL	32–36 g/dL
WBC	11.2 × 10^9^/L	4.0–10.0 × 10^9^/L
Neutrophils	86%	40%–75%
Lymphocytes	11.9%	20%–45%
Platelets	220 × 10^9^/L	150–400 × 10^9^/L
ESR	40 mm/h	0–25 mm/h
**Coagulation**		
PT (patient/control)	14 s/12 s	11–13.5 s
INR	1.16	0.9–1.2
APTT (patient/control)	34 s/32 s	25–40 s
D-dimer	0.073 mg/L	< 0.5 mg/L FEU
**Biochemistry**		
Albumin	15.5 g/L	35–50 g/L
AST	22 U/L	0–40 U/L
ALT	28 U/L	0–40 U/L
Alkaline phosphatase	48 U/L	44–147 U/L
**Electrolytes**		
Sodium	136 mmol/L	136–145 mmol/L
Potassium	4.2 mmol/L	3.5–5.1 mmol/L
Chloride	96 mmol/L	98–106 mmol/L
Bicarbonate	20 mmol/L	23–29 mmol/L
**Iron Studies**		
Ferritin	6 ng/mL	15–150 ng/mL
Serum iron	28 μg/dL	60–170 μg/dL
TIBC	465 μg/dL	240–450 μg/dL
**Celiac Serology**		
Anti-tTG IgG	22.8 U/mL	< 10 U/mL
Anti-tTG IgA	1.6 U/mL	< 10 U/mL
Total Serum IgA	4 mg/dL	70–400 mg/dL

RBC, Red Blood Cell count; MCV, Mean Corpuscular Volume; MCHC, Mean Corpuscular Hemoglobin Concentration; WBC, White Blood Cell Count; ESR, Erythrocyte Sedimentation Rate; PT, Prothrombin Time; INR, International Normalized Ratio; APTT, Activated Partial Thromboplastin Time; AST, Aspartate Aminotransferase; ALT, Alanine Aminotransferase; TIBC, Total Iron-Binding Capacity; Anti-tTG, anti-tissue transglutaminase; IgA, immunoglobulin A; IgG, immunoglobulin G.

Upper gastrointestinal endoscopy revealed a 3 × 3 cm ulcerated, infiltrative mass in the lower third of the esophagus, approximately 35 cm from the incisors, causing near-total luminal obstruction (Fig. 1). Duodenal biopsy showed villous atrophy, crypt hyperplasia, and increased intraepithelial lymphocytes, consistent with Marsh 3 celiac disease. Biopsy from the esophageal lesion confirmed moderately differentiated keratinizing squamous cell carcinoma with keratin pearls and pleomorphic hyperchromatic nuclei (Fig. 2).

**Figure 1:**
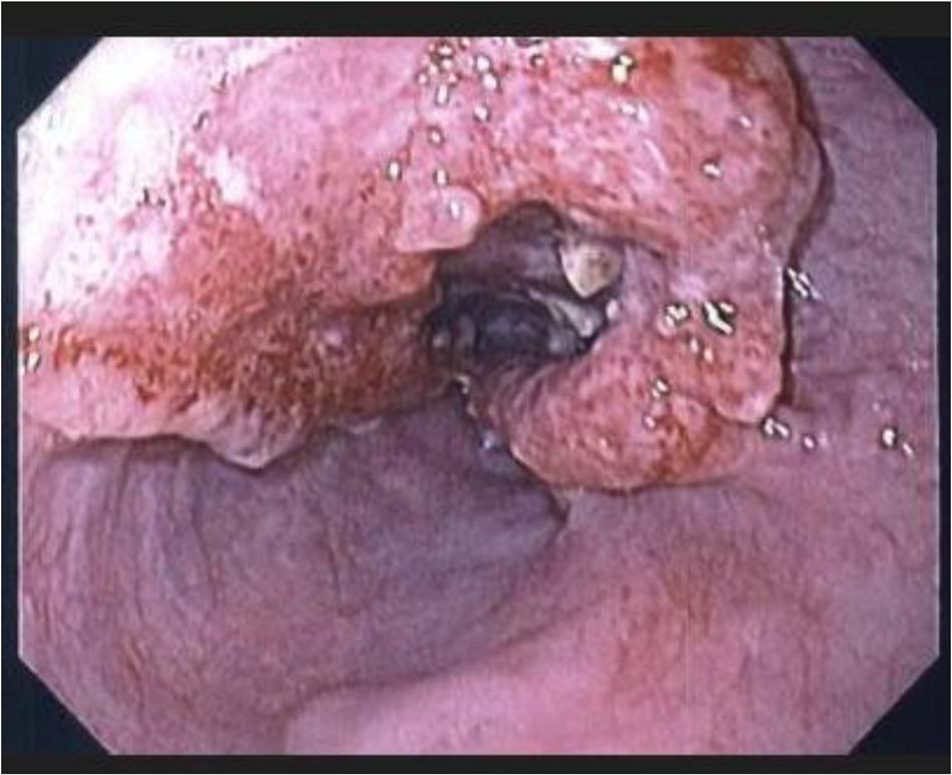
Endoscopic image, showing an ulcerated and partially obstructing mass in the distal third of the esophagus with friable mucosa and irregular, nodular surface, consistent with esophageal squamous cell carcinoma (ESCC). The lesion involves a portion of the circumference without forming a complete ring.

**Figure 2:**
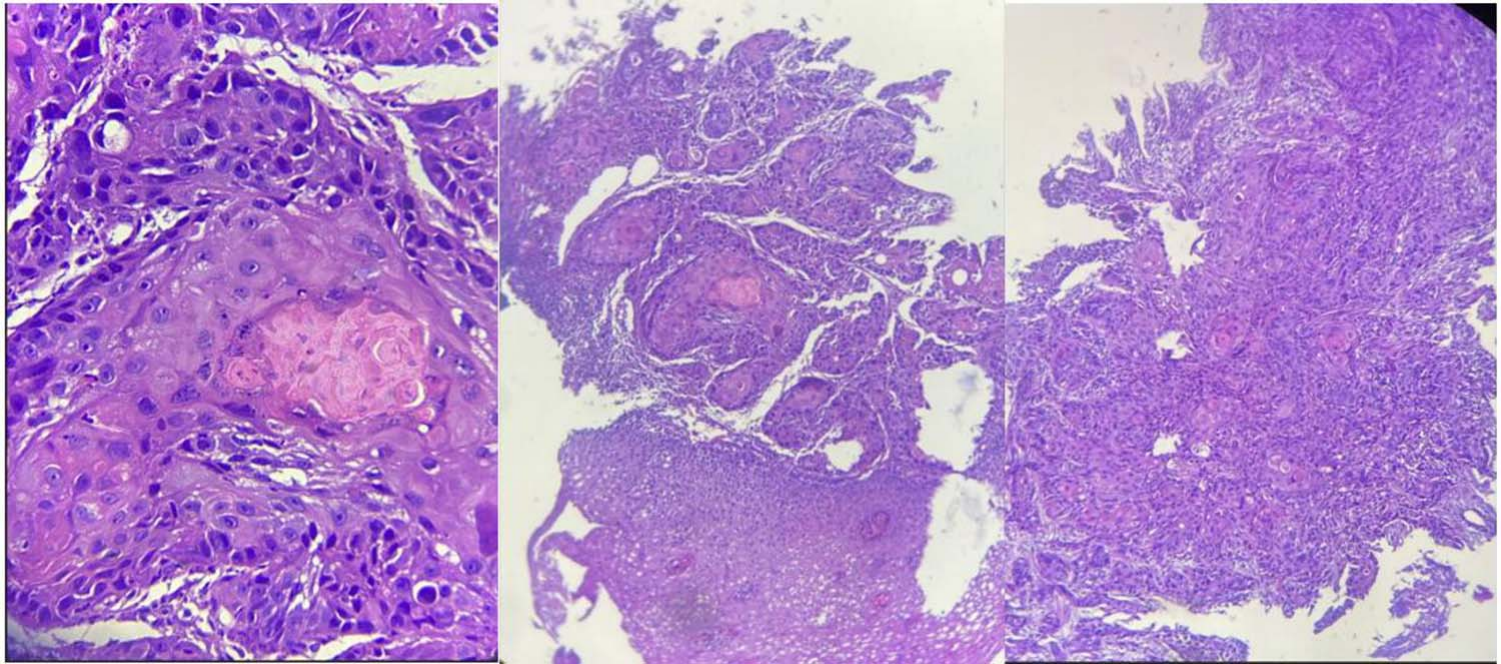
Histopathological sections (H&E stain, ×40 and ×100), showing nests of atypical squamous cells with eosinophilic cytoplasm, keratin pearls (whorled keratin centers), pleomorphic hyperchromatic nuclei, and infiltrative architecture. Features are consistent with moderately differentiated keratinizing squamous cell carcinoma.

The initial contrast-enhanced CT and PET imaging were performed at the time of diagnosis in January 2025, which demonstrated heterogeneously enhancing mural thickening of the distal esophagus from T6 to T9, involving the gastroesophageal junction and adjacent gastric cardia, fundus, and lesser curvature. FDG-avid lymphadenopathy was seen in the gastrohepatic ligament and left para-aortic region, without distant organ metastasis (Fig. 3A–D). Based on endoscopic, imaging, and pathological assessment, the epicenter of the lesion appeared to be in the distal esophagus, approximately 35 cm from the incisors, with contiguous distal extension across the gastroesophageal junction into the adjacent gastric cardia, fundus, and lesser curvature. Therefore, the tumor was managed anatomically as a primary distal esophageal squamous cell carcinoma, extending distally.

**Figure 3:**
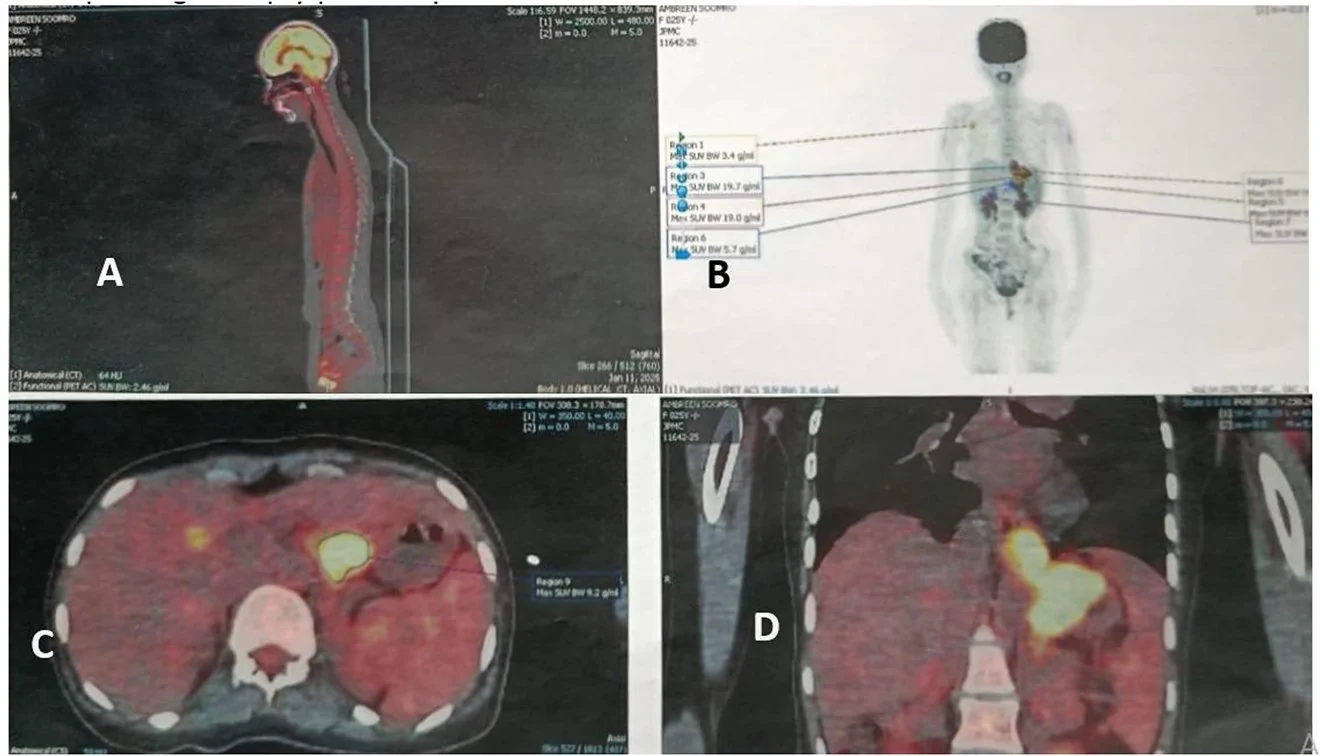
FDG positive PET/CT of distal esophageal carcinoma and nodal staging. (A and B) Whole-body maximum intensity projection (MIP) FDG PET sagittal image demonstrating multiple hypermetabolic foci: Distal esophageal, gastrohepatic ligament, and left Para-aortic area. (C) Axial fused PET/CT image at the level of the gastroesophageal junction showing intense FDG uptake within the distal esophageal mass, demonstrating partial circumferential mural mass in the distal third of the esophagus, causing near-total luminal obstruction. (D) Coronal fused PET/CT image demonstrating intense FDG uptake within the distal esophageal mass, corresponding to biopsy-proven squamous cell carcinoma.

The patient was started on supportive therapy, including proton-pump inhibitors, antibiotics, nutritional support, and feeding jejunostomy. Following multidisciplinary discussion among gastroenterology, cardiothoracic surgery, and oncology teams, upfront curative surgery was deferred because of the locally advanced disease extent and severe malnutrition. Neoadjuvant chemoradiotherapy was therefore planned with the intention of tumor downstaging, nutritional optimization, and subsequent surgical reassessment if the disease became resectable. She received weekly paclitaxel and carboplatin with concurrent external-beam radiotherapy. During treatment, she developed mucositis and transient chemotherapy-induced neutropenia, which improved with supportive care and granulocyte colony-stimulating factor. After completion of all 28 planned radiotherapy sessions and 12 of 16 planned weekly chemotherapy cycles, a follow-up contrast-enhanced CT performed in June 2025, approximately five months after the initial PET/CT, showed interval progression of the distal esophageal mass with persistent regional lymphadenopathy and no distant metastasis.

At last follow-up, she remained hemodynamically stable with partial symptomatic relief on proton-pump inhibitors, analgesics, and enteral nutrition. In view of interval disease progression despite chemoradiotherapy and persistent severe malnutrition, the multidisciplinary team did not consider curative esophagectomy feasible. Further systemic and palliative options are being discussed with her and her family, with emphasis on symptom control and quality of life.

## Discussion

This case is unusual because ESCC occurred in a young woman with longstanding celiac disease, absence of conventional risk factors, and a tumor located in the distal esophagus. In Pakistan, ESCC is typically diagnosed at a substantially older age, with a reported mean age of approximately 49 years [5]. Furthermore, distal esophageal tumors are more commonly adenocarcinomas, classically linked to Barrett’s esophagus and gastroesophageal reflux disease [7]. The presence of keratinizing ESCC in the distal third, therefore, represents an uncommon clinicopathological presentation.

Although celiac disease is classically associated with enteropathy-associated T-cell lymphoma and small bowel adenocarcinoma, emerging evidence also suggests a specific association with esophageal squamous cell carcinoma. In a nationwide Dutch pathology database study, van Gils et al. reported that celiac disease was associated with an increased relative risk of esophageal squamous cell carcinoma (RR 3.5, 95% CI 2.1–5.8), with a stronger association observed among female patients (RR 5.9, 95% CI 3.3–10.3) [8]. The association between celiac disease and malignancy may be related to chronic inflammation, immune dysregulation, malabsorption, and nutritional deficiencies [9]. In this patient, severe malnutrition, hypoalbuminemia, microcytic anemia, and selective IgA deficiency may have contributed to impaired mucosal repair and immune surveillance. Selective IgA deficiency has been reported in approximately 2%–18% of patients with celiac disease and may further compromise mucosal immunity [10]. Although causality cannot be established from a single case, these factors may have contributed to a pro-oncogenic environment.

This case also highlights the importance of early endoscopic evaluation in patients with celiac disease who develop alarm symptoms. Our patient developed rapidly progressive dysphagia, weight loss, and melena within a short period. Nutritional optimization through feeding jejunostomy was essential before chemoradiotherapy. However, despite completing radiotherapy and most planned chemotherapy cycles, follow-up imaging showed disease progression, suggesting aggressive tumor biology and the adverse impact of malnutrition on treatment response.

This report has limitations. It describes a single case with a relatively short follow-up of 8 months, and molecular profiling or germline testing was not performed. Therefore, no firm conclusion can be made regarding causality or shared genetic mechanisms between celiac disease and ESCC. Larger studies are needed to validate this possible association.

In conclusion, distal keratinizing ESCC in a young woman with celiac disease and no conventional risk factors is exceptionally rare. New-onset dysphagia, weight loss, or gastrointestinal bleeding in patients with celiac disease should prompt early endoscopic evaluation, regardless of age or traditional cancer risk factors.

## References

[1] Sung H, Ferlay J, Siegel RL. et al. Global cancer statistics 2020: GLOBOCAN estimates of incidence and mortality worldwide for 36 cancers in 185 countries. CA Cancer J Clin 2021;71:209–49. 10.3322/caac.21660 33538338

[2] Arnold M, Soerjomataram I, Ferlay J. et al. Global incidence of oesophageal cancer by histological subtype in 2012. Gut 2015;64:381–7. 10.1136/gutjnl-2014-308124 25320104

[3] Abnet CC, Arnold M, Wei WQ. Epidemiology of esophageal squamous cell carcinoma. Gastroenterology 2018;154:360–73. 10.1053/j.gastro.2017.08.023 PMC5836473 28823862

[4] Han Y, Chen W, Li P. et al. Association between coeliac disease and risk of any malignancy and gastrointestinal malignancy: a meta-analysis. Medicine (Baltimore) 2015;94:e1612. 10.1097/MD.0000000000001612 PMC4635766 26402826

[5] Butt N, Soomro SA, Haider G. et al. Epidemiological and clinical characteristics of esophageal carcinoma: an experience from tertiary care hospital of Karachi, Pakistan. Pak. J Med Sci 2024;40:55–9. 10.12669/pjms.40.1.7419 PMC10772445 38196475

[6] Gagnier JJ, Kienle G, Altman DG. et al. The CARE guidelines: consensus-based clinical case reporting guideline development. BMJ Case Rep 2013;2013:bcr2013201554. 10.1136/bcr-2013-201554 PMC3822203 24155002

[7] Rustgi AK, El-Serag HB. Esophageal carcinoma. N Engl J Med 2014;371:2499–509. 10.1056/NEJMra1314530 25539106

[8] Van Gils T, Nijeboer P, Overbeek LIH. et al. Risks for lymphoma and gastrointestinal carcinoma in patients with newly diagnosed adult-onset celiac disease: consequences for follow-up. United European Gastroenterol J 2018;6:1485–95. 10.1177/2050640618800540 PMC6297918 30574319

[9] Green PH, Jabri B. Coeliac disease. Lancet 2003;362:383–91. 10.1016/S0140-6736(03)14027-5 12907013

[10] Cataldo F, Marino V, Ventura A. et al. Prevalence and clinical features of selective immunoglobulin a deficiency in coeliac disease: an Italian multicentre study. Gut 1998;42:362–5. 10.1136/gut.42.3.362 PMC1727042 9577342

